# Anniversary Discourse before the N. Y. Academy of Medicine

**Published:** 1851-03

**Authors:** 


### ARTICLE III.

*Anniversary Discourse before the New York Academy of Medicine.* By JOSEPH M. SMITH, M. D., 56 pp. octavo, 1851.

*Introductory Address delivered before the Students and Trustees of the New York Medical College.* By HORACE GREEN, A. M., M. D., President of the Faculty, and Prof. of Theory and Practice of Medicine. (From the author.)

*Introductory Address to the Class of the Pennsylvania College, session*, 1850-51. By WASHINGTON L. ATLEE, M. D., Prof. of Medical Chemistry. (From the author.)

The discourse of Dr. Smith is devoted to the consideration of the peculiar mental phenomena of the soldier, and is rich in historical reminisences illustrating the subject. Striking incidents in the lives of celebrated warriors, that class of men who have in all ages claimed the largest share of public consideration, could scarcely fail to interest; and as these are judiciously introduced to show the different mental conditions of the soldier under varied circumstances, the author has made a very interesting discourse.

The lecture of Dr. Green was given at the opening of a new Medical College in the City of New York, and will be generally regarded with interest, as laying down the course the new school has proposed to pursue. With the finest building of any Medical School in the great State of New York, and a faculty embodying much talent and energy, and the regular and systematic course proposed by Dr. Green, the N. Y. Medical College can scarcely fail of a successful career.

The lecture of Dr. Atlee is well conceived, being advice to those entering upon a course of instruction in the institution; giving them rules for their guidance in the acquisition of the knowledge they seek, and sound admonition in reference to the dangers of yielding to the temptations to immorality; and commending the beauty of principles of virtue and piety combined with learning and skill in the profession. E.

